# Spinal arachnoid diverticula and constrictive myelopathy in dogs: different conditions or a spectrum of spinal meningeal disease in veterinary and human medicine?

**DOI:** 10.3389/fvets.2026.1783066

**Published:** 2026-07-06

**Authors:** João Miguel De Frias, Sadaquate Khan, Cecilia Rohdin, Sofie F. M. Bhatti, Steven De Decker

**Affiliations:** 1 Hospital for Small Animals, The Royal (Dick) School of Veterinary Studies, University of Edinburgh, Edinburgh, United Kingdom; 2 Department of Neurosurgery, Royal Infirmary of Edinburgh, Edinburgh, United Kingdom; 3 AniCura Djursjukhuset Albano, Danderyd, Sweden; 4 Small Animal Department, Faculty of Veterinary Medicine, Ghent University, Merelbeke-Melle, Belgium; 5 Department of Clinical Science and Services, The Royal Veterinary College, University of London, London, United Kingdom

**Keywords:** meningeal fibrosis, one health, spinal meningeal disease, subarachnoid cyst, translational research

## Abstract

Different conditions affecting the spinal meningeal layers have been reported in human and veterinary medicine. In veterinary medicine, two separate conditions have been reported. Spinal arachnoid diverticula (SAD) have been well documented for decades, while constrictive myelopathy (CM) in pugs was identified more recently. However, the distinction between SAD and CM in dogs can be challenging. There is a lack of understanding about the pathophysiology of these puzzling conditions, which impacts the ability to select the most appropriate treatment. In humans, four different spinal meningeal diseases are described, including intradural or extradural spinal arachnoid cysts, spinal arachnoid webs, spinal adhesive arachnoiditis, and idiopathic spinal cord herniation. Similar challenges to veterinary medicine are also encountered in human medicine. This brief comparative review aims to emphasize the parallels and obstacles found in both veterinary and human medicine and to explore potential future developments. The term ‘spinal meningeal adhesive disease’ is proposed as a unified term in veterinary medicine for pathologies affecting the meninges of the spinal cord (SAD and CM), potentially causing progressive clinical signs of myelopathy.

## 1 Introduction

Since the first description of a leptomeningeal cyst in a dog over 50 years ago (1), several publications have described similar spinal meningeal pathologies in dogs. Different terminologies have been used for the same spinal condition: meningeal cyst (2, 3), arachnoid pseudocyst (4), arachnoid cyst (5–10), intradural arachnoid cyst (11), subarachnoid cyst (12, 13), subarachnoid diverticulum (14–19), and intra-arachnoid diverticulum (20, 21). Currently, the most widely utilized term is spinal arachnoid diverticulum (SAD) (22–27). Cats diagnosed with SAD have been reported (15, 28–36), as has one horse (37). The classification of this disease has changed from an arachnoid ‘cyst’ to a diverticulum, given the absence of an enclosed structure with an epithelial cell lining in histopathological analyses (21). SAD affects the cervical, thoracic, and lumbar regions.

More recently, an additional spinal meningeal condition was identified. Constrictive myelopathy (CM) was first described by Fisher et al. as a distinctive spinal pathology in pugs, affecting the thoracolumbar region (38). CM has also been referred to as meningeal fibrosis (39) and pia-arachnoid fibrosis (40, 41); however, these features have also been identified in SAD. Diagnostic imaging criteria have been proposed for thoracolumbar CM in dogs (42). However, the diagnostic distinction between thoracolumbar SAD and CM remains challenging. This raises the pertinent question: are thoracolumbar SAD and CM on the same disease spectrum? In a study by Rohdin et al., an arachnoidal diverticulum formation was identified by MRI and concomitantly on histopathology in over half of the pug population with a histopathologic diagnosis of meningeal fibrosis (39).

This review aims to briefly describe spinal meningeal diseases in veterinary and human medicine, highlight the similarities and challenges, propose a unified terminology, and discuss possible future directions. This manuscript is not meant to be an exhaustive review of the literature. The intent is to underscore the importance of collaboration between human and veterinary medicine in addressing similar pathologies.

## 2 Veterinary medicine

### 2.1 Spinal arachnoid diverticula: current understanding and remaining questions

Spinal arachnoid diverticula (SAD) are defined as a focal enlargement of the subarachnoid space filled with cerebrospinal fluid that causes spinal cord compression (21, 22, 26). Clinical signs most commonly present as progressive, non-painful proprioceptive ataxia with a variable degree of motor deficits. A low percentage (19%) of dogs can present with spinal hyperesthesia (23). Fecal and/or urinary incontinence can also be present in dogs with SAD (8, 12, 23, 43). This pathophysiology is likely related to dysfunction of the dorsally located ascending sensory pathways in the spinal cord (8, 19, 23, 26, 44). Urinary dysfunction is believed to be a consequence of an upper motor neuron lesion, and it is characterized by an inability to produce an uninterrupted flow of urine, with continued dribbling after urination, which is consistent with urethral dyssynergia (8, 19). Fecal incontinence is characterized by an inability to stop the urge to evacuate, resulting in accidental defecation that may go unnoticed (8, 19). The same classification by localization for spinal arachnoid cysts (SACs) in humans has been proposed for SAD in dogs (21). Intradural SACs (type III) show features similar to those seen in dogs (21, 45). Furthermore, type II SACs, which are extradural meningeal cysts with involvement of the nerve root (Tarlov or perineural cysts), have also been identified in dogs (21, 45, 46). A breed predisposition to SAD has been well established in dogs. In the cervical region, it is most commonly reported in Rottweilers and pugs, while in the thoracolumbar region, it is most commonly reported in pugs and French bulldogs (21–23, 26, 27). Although SAD is considered a multifactorial disease, the distinction between likely primary (idiopathic) and secondary (acquired due to another spinal disease) remains challenging (19, 22, 26). The reason for this difficulty is mainly related to the wide range of presentations because of the different rates at which diverticula fill in each case (2, 7, 8, 12, 13, 19, 20, 22, 26, 29, 43, 47–51). Furthermore, although a tendency to present clinical signs at a younger age is seen in presumed congenital SAD cases, the high prevalence of concurrent or adjacent neurological disease at the same level in predisposed breeds, such as pugs and French bulldogs, makes this distinction difficult (2, 7, 8, 12, 13, 19, 20, 22, 26, 29, 43, 47–51). A distinctive ‘teardrop’ shape on diagnostic imaging is found in SAD cases (22, 26). Currently, the diagnosis of SAD is generally made with magnetic resonance imaging (MRI). The addition of T2-W sequences that allow reconstruction, such as 3D constructive interference in steady state (3D-CISS), alongside sagittal sequences for the detection of CSF columns, such as half-Fourier acquisition single-shot turbo spin-echo (HASTE), remarkably improves the detection of SAD cases (52, 53). Better identification of SAD with MRI allows for recognition of different SAD conformations (54). The presence of concurrent syringomyelia has also been more accurately detected and related to the location of SAD (54).

There is medical or surgical treatment available for SAD. There is some evidence that a better outcome is seen in surgically treated cases (27). Described surgical techniques include (hemi)laminectomy or ventral slot, followed by durotomy, durectomy, fenestration, and dural marsupialization, with or without adhesiolysis and vertebral stabilization (20, 22, 26, 27, 55). Recurrence of clinical signs following surgery, not necessarily of SAD reformation, has been reported (8, 25, 31, 32, 36). To overcome this, the placement of an intrathecal shunt has been suggested (20, 56). Recently, a lower rate of recurrence has been identified with this technique compared to durotomy alone (14.3% vs. 41.7%) (57). However, a higher recurrence rate was observed in pugs, regardless of treatment (57). This highlights the need to better understand the pathophysiology prior to making treatment recommendations, as different approaches may be necessary.

### 2.2 Constrictive myelopathy: current understanding and remaining questions

Constrictive myelopathy (CM) is characterized by the presence of a fibrotic band surrounding the spinal cord, which leads to focal spinal cord constriction (38, 39, 42). CM has been identified in the caudal thoracolumbar region, typically between T11 and L1 (38, 42). Similar to SAD, CM presents with clinical signs of myelopathy (Table 1), although a higher prevalence (over 60% of cases) of urinary/fecal incontinence is reported (42). Given the association of CM with pugs and the predisposition of this breed to other chronic spinal cord disorders (such as SAD and intervertebral disk protrusion), the term “pug dog thoracolumbar myelopathy” (PTM) has been growing in popularity (58, 59). CM is generally considered a disease of older dogs (38, 39, 42). More recently, CM has also been described in West Highland White Terriers (60). CM was originally proposed to develop secondarily to vertebral micro-instability in the highly mobile caudal thoracic region secondary to caudal articular process dysplasia (CAPD) (41, 42). However, CAPD is a common malformation in pugs, reported in up to 91.2 to 97% of neurologically normal pugs (61, 62). Moreover, CM has been reported in pugs with other vertebral malformations, such as hemivertebrae, and in pugs without concurrent vertebral anomalies (39, 42, 63). These findings call into question the causative relationship between CAPD and CM. More recently, another possible culprit has been suggested. Chronic spinal cord insults, such as vertebral dynamic instability or disk herniations, can lead to lympho-histiocytic inflammation with astrocyte activation (64). Further supporting this hypothesis, histopathology of pugs with CM revealed lympho-histiocytic inflammation, including encephalitis, myelitis, and pachymeningitis with plaque-like mineralization (39). Lympho-histiocytic inflammation is also a predominant feature of necrotizing meningoencephalitis in pugs (65). The production of anti-GFAP autoantibodies has been speculated to be involved in the formation of fibrosis, which leads to adhesions (39, 64). Moreover, the NME risk haplotype has been found to be associated with an earlier onset of clinical signs in male pugs (59). The significance of the presence of inflammatory features in CM has not been fully established and warrants careful consideration when investigating this condition. The best treatment option for CM remains to be identified. Similar surgical techniques to the ones described for SAD, with the addition of stabilization, have been suggested (41, 66). Although an initial improvement was reported, the long-term outcome remains poor (39, 66).

**Table 1:** Comparison between clinical features and advanced imaging findings in human and veterinary spinal meningeal pathologies.

		Age of onset	Sex distribution	Clinical signs	Spinal level	Lesion shape	Arachnoid bridge	Spinal disease associated with lesions	Intramedullary signal changes	Gadolinium enhancement
Veterinary medicine	SAD (23, 27)	Wide range (18 weeks to 13 years).	Male predominance	Proprioceptive ataxia (92.6%). Hypermetria (21.3%). Hyperesthesia (18.9%). Fecal and/or urinary incontinence (8.2%). Paresis (4.9%).	All levels of the vertebral column: 50% cervical and 50% thoracolumbar.	Subarachnoid space-filling lesion, with a teardrop shape.	Arachnoid adhesions can sometimes be visualized.	Concurrent spinal disorders significantly associated with French Bulldogs and Pugs.	Syringomyelia: Cervical – up to 58.3% of dogs. Thoracolumbar – up to 82% of dogs.	None
	CM (42)	Older dogs (over 7 years old).	Male predominance	All cases presented with pelvic limb ataxia and paresis (50% with lateralized signs). Fecal and/or urinary incontinence (over 60%). Spinal hyperesthesia (15.4%).	Thoracolumbar.	Focal, irregular, stellate, marginal spinal cord appearance.	Multiple adhesions present.	Presence of caudal articular process dysplasia. Bilateral ventrolateral extradural lesion.	Intramedullary signal changes.	Circumferential enhancement.
Human medicine	SACs (78)	Intradural: mean age 48 years. Extradural: mean age 39 years.	No clear sex predominance	Spinal pain (69%) Gait ataxia (69%) Hypoesthesia/Dysesthesia (50%) Sphincter dysfunction (27%)	Predominantly thoracic (85%). Lumbar (5%). Cervical (2.8%).	Subarachnoid space-filling lesion with a scalpel sign. Cinematic MRI improves diagnosis.	A circumferential capsule may be seen.	Not described.	Syringomyelia in 40% of cases.	None
	SAWs (75)	Mean age 56 years.	Male predominance	Sensation disturbances (68.5%) Pain (64.6%) Motor weakness (60.2%) Sphincter dysfunction (22.1%)	Predominantly thoracic region. Cervical (0.58%).	Subarachnoid space-filling lesion with a scalpel sign.	Arachnoid adhesions can be visualized.	Trauma (10%). Surgery (4%). Multiple sclerosis (2%).	Syringomyelia in 75% of cases.	None
	SAA (77)	Mean age 44 years.	No clear sex predominance	Motor weakness (78.6%) Abnormal nerve sensations (39.6%) Spinal pain (37.8%) Sphincter dysfunction (40.6%)	All levels of the vertebral column: Thoracic (49%). Lumbar (28.7%). Cervical (15.8%)	Asymmetrical and irregular compression of the spinal cord with subarachnoid space widening (60% are multifocal).	Spinal cord or nerve root clumping/tethering/thickening.	Trauma (22.7%), infection (17.7%), surgery (15.4%), hemorrhage (13.5%), Chiari malformation type I (5.7%), spinal anesthesia (5.4%), myelography (4.5%), herniated disks and spinal stenosis (3.5%), spinal injections (3%).	Syringomyelia in 30%.	Can be present in nerve roots, the spinal cord, or adhesions.
	SCH (124)	Mean age 40 years.	Female predominance	Wide spectrum of symptoms: motor and sensory disturbances (e.g., loss of pain and temperature), myoclonus, bladder dysfunction, and positional radicular pain.	Thoracic spine	Ventral dural defect causing a C-shaped kink of the spinal cord.	None	Focal scalloping of the posterior margin of the vertebral body.	None	None

Data were collected from the largest and most recent cohorts of case studies or systematic reviews.

## 3 Human medicine

Spinal meningeal diseases (SMDs) have recently been proposed in human medicine as terminology to refer to pathologies that affect the meningeal layers surrounding the spinal cord and cause progressive neurological clinical signs (67). There is considerable overlap in the anatomical features and imaging findings (‘scalpel-blade sign’) between different SMDs (67–70). One of the difficulties in investigating SMDs is the rarity of these conditions (71–75). However, it is still possible that SMDs have been underreported due to the lack of advanced MRI sequences and techniques, such as multiplanar reconstruction (MPR), in the past (76, 77). SMDs have been differentiated into four main different pathologies: intradural or extradural spinal arachnoid cysts, spinal arachnoid webs, spinal adhesive arachnoiditis, and idiopathic spinal cord herniation (67).

### 3.1 Spinal arachnoid cysts

Spinal arachnoid cysts (SACs) are defined as cerebrospinal fluid-filled enclosed structures lined by an arachnoid membrane and fibrous tissue (45, 78–84). SACs are primarily found dorsally in the subarachnoid space and in the thoracic region (70%), followed by the lumbar (25%) and cervical (5%) regions (78, 85, 86). SACs were traditionally classified according to the localization in the vertebral canal (45), but more recently a classification based on presumed etiology has been proposed (87, 88). Regarding localization, SACs are divided into type I (extradural meningeal cysts without involvement of the nerve root), type II (extradural meningeal cysts with involvement of the nerve root), and type III (intradural meningeal cysts) (45). SACs are classified according to etiology as primary (or idiopathic) and secondary (or acquired) following spinal disease or surgery (87, 88). Interestingly, primary SACs are typically found at the thoracic level, while secondary SACs are found in all spinal regions and are generally related to more severe neurological deficits (87, 88). Extradural SACs have recently been reclassified as dural diverticula (87, 88). If this dural defect is near a nerve root sleeve, it is also named a Tarlov cyst, root sleeve cyst, or perineural cyst (87–89). The main theory for the initial formation of SACs is that they originate from an arachnoid herniation for extradural SACs or from the dissection of the posterior intradural spinal ligament, also known as the *septum posticum*, for intradural SACs (90, 91). A one-way ball valve mechanism with communication between the cyst and the subarachnoid space, leading to cyst enlargement, has been demonstrated with intraoperative ultrasound (92). Therefore, SACs are better defined as diverticula, given their communication with the subarachnoid space (93–95). An attempt at a unified approach by using the term “arachnoid diverticula” has been made by Fortuna et al. (93). Furthermore, another explanation for the origin of SACs has also been provided that involves arachnoid granulations (93, 96). These structures are found at the margin of the subarachnoid space adjacent to the medial pole of the dorsal root ganglion (93, 96). If these arachnoid granulations become hypertrophic and entrapped, this causes a restriction in the variations of CSF hydrodynamics, leading to diverticulum formation (93, 96). Acquired SACs are considered uncommon and represent only 13% of reported cases (79). Underlying diseases include historical trauma, iatrogenic causes (i.e., previous spinal surgery or lumbar CSF tap), hemorrhage, and meningitis (79). Interestingly, intradural SACs commonly cause pain, particularly when associated with syringomyelia (in up to 84% of the patients) (78). Other clinical signs of myelopathy, as expected for progressive spinal cord compression, are described in Table 1. The preferred treatment for symptomatic SACs is surgery (78, 81, 97). However, observational monitoring has also been described (98). This approach is reserved for asymptomatic patients with smaller cysts (98). However, 39% of these patients eventually required surgery (98). Described surgical techniques for SACs include cyst excision, fenestration, and cysto-subarachnoid shunting (78, 81, 97). Excision of SACs, with removal of adhesions, provides the lowest risk of recurrence (78, 81, 97). Factors associated with poor recovery include a marked compression of the spinal cord by the cyst and a longer duration of paresis (99).

### 3.2 Spinal arachnoid webs

**Figure 1:**
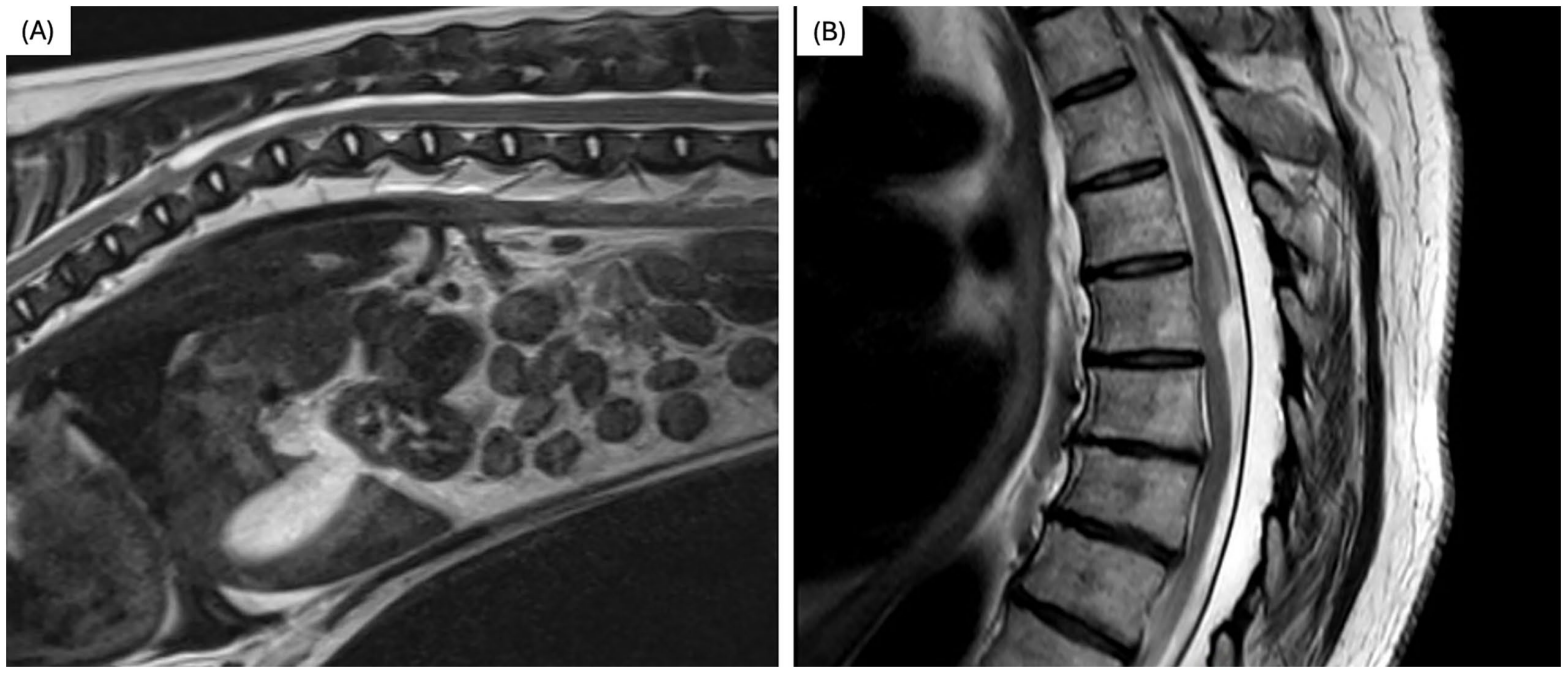
Examples of magnetic resonance imaging of spinal meningeal pathologies in veterinary and human medicine in a T2-weighted sequence sagittal view. **(A)** A 9-month-old male pug with a caudal tethered spinal arachnoid diverticulum at the T9/T10 level. **(B)** A 64-year-old women with a spinal arachnoid web at the T5/T6 level. The imaging similarities between the ‘teardrop’ **(A)** and ‘scalpel-blade’ **(B)** shapes of both cases should be noted.

Spinal arachnoid webs (SAWs) are a distinctive pathology from SACs (71, 100). SAWs are characterized by the presence of a thickened band of arachnoid fibers that extends from the pial surface of the dorsal aspect of the spinal cord (71, 100, 101). SAWs normally occur at the thoracic level and can have an insidious course with a presentation ranging from asymptomatic to severe myelopathy (71, 100, 101). SAWs have also rarely been described in the cervical region (102). The pathophysiology of SAWs is not completely understood. The fibroconnective adhesions found in SAW cases could be the result of known or insidious inflammation of the arachnoid trabeculae or the intermediate ligaments of the leptomeninges (72, 75, 100, 103). Previous spinal injury or surgery causing scarring, infections, or spinal hemorrhage has been linked as a possible risk factor for the formation of SAWs (70, 72, 75, 90, 104). However, only a small proportion of patients have been found to have these risk factors in recent systematic reviews (72, 75). Furthermore, SAWs could be the consequence of a ruptured SAC or represent a precursor to this disease (68, 105, 106). Further supporting this link, histopathological studies of SAWs have revealed fibrous connective (collagenous) tissue associated with meningothelial cells and arachnoid cells, which is similar to what has been reported for SACs (75, 78, 107). The established sagittal imaging finding for SAWs is the formation of a “scalpel-blade” sign in the subarachnoid space, as visualized in T2-weighted images (Figure 1) (67, 71, 108). However, the “scalpel-blade” sign can also be found in other spinal meningeal diseases (69, 72, 100, 109–112). It is well established that the treatment of choice for symptomatic SAWs is surgery (71, 72, 75, 100, 101). Surgical techniques described include complete adhesiolysis and arachnoid web excision (71, 72, 75, 100, 101) or bypassing CSF flow with a shunt or stent (72). Recurrence is rare, and the long-term outcome appears favorable (71, 72).

### 3.3 Spinal adhesive arachnoiditis

Spinal adhesive arachnoiditis (SAA) is an uncommon disease caused by chronic inflammation of the arachnoid layer. This leads to hyalinization, thickening, and adhesion of the nerve roots and/or tethering of the spinal cord. SAA can occur secondary to infections, trauma, tumors, genetics, and iatrogenically (e.g., after myelographic studies) (67, 77, 113). Interestingly, SAA in the thoracic region appears to be more likely to be caused by mechanical injury, while SAA in the cervical region is more likely secondary to inflammatory diseases (77). In some cases, end-stage dystrophic calcification can occur, and this condition is known as *arachnoiditis ossificans*, which is associated with a very poor prognosis (114, 115). The most common features seen on MRI include the formation of an arachnoid cyst, followed by abnormal nerve roots/spinal cord tethering (spinal arachnoid web) with variable degrees of contrast enhancement and the presence of syringomyelia (116, 117). Another MRI feature particularly associated with SAA is meningeal contrast enhancement (67). This MRI feature is not seen with SACs and SAWs. Although multiple arachnoid cysts or webs/adhesions are more common in SAA, focal pathology can also be present (70, 116, 118–120). Therefore, SAA could still be misdiagnosed as SAC or SAW (67). Although early pharmacological or surgical intervention has been described for SAA, the outcome is poor (119, 120). Pharmacological treatment consists of systemic immunotherapy or epidural corticosteroid injections, which can be partially successful in the early stages of SAA (77, 119–121). Surgical techniques include either direct arachnoid adhesion dissection or CSF shunting. While arachnoid adhesion dissection can initially be effective for focal SAA, syringoperitoneal shunting achieves more sustainable, long-term results by controlling the syringomyelia associated with SAA (122). In one study, the addition of spinal fusion, with or without CSF shunting, offered an 80% success rate, with an average follow-up period of more than 4 years (123). The rationale behind spinal fusion was related to the impact of spinal mobility on the development and/or manifestation of SAA (123). This study suggested that, regardless of the underlying causes and concurrent imaging findings, it is reasonable to attempt a more holistic treatment for all possible causes (123). Nevertheless, SAA is considered a chronic and highly debilitating disease with no cure (77, 119–121). Therefore, the treatment for SAA is considered symptomatic.

### 3.4 Idiopathic spinal cord herniation

Idiopathic spinal cord herniation (SCH) is a very rare condition characterized by a herniation of the spinal cord through a ventral dural or arachnoidal defect into the adjacent epidural space, with no obvious cause identified for this defect (124–129). The most common site of an ISCH is within the thoracic region, specifically between T4 and T7, which is the site of physiological kyphosis (124, 125). Although the etiology of idiopathic SCH is not completely understood, it has been speculated that it occurs secondarily to a previous trauma, disk herniation, a ventral congenital defect, or secondary to a previous inflammatory disease (124–127, 130). However, it has been proposed that idiopathic SCH is formed secondarily to an intradural SAC or duplication of the ventral dura (77, 131–134). Treatment is surgical with intradural adhesiolysis combined with dural defect repair or with dural patch placement (127). Surgery is considered the treatment modality of choice, with a good prognosis reported (127, 135, 136).

## 4 Discussion

Spinal meningeal pathologies in veterinary medicine share many similarities with those in human medicine. However, a definitive pathophysiology is not well established. Both idiopathic and secondary etiologies have been described, with idiopathic conditions remaining particularly puzzling, as their origin remains difficult to ascertain. Moreover, there is a striking resemblance in imaging features among humans, dogs, and cats. The ‘scalpel-blade’ and ‘teardrop’ MRI descriptions share the same visual appearance (Figure 1). Furthermore, there is a considerable overlap of imaging features such as: (1) the presence of adhesions, (2) conformation, and (3) spinal cord parenchymal changes, such as the presence of syringomyelia or myelomalacia. However, idiopathic spinal cord herniation has not yet been described in veterinary medicine.

Despite the fact that imaging characteristics of CM in pugs have been reported (42), differentiating thoracolumbar SAD and CM in pugs can still be challenging. While it is possible that SAD and CM in pugs are different diseases, it cannot be excluded that they represent different stages of the same spinal meningeal pathology. In human medicine, SACs have been proposed to be a variant of SAWs in the thoracic region (68). This comparison seems to be more applicable to the thoracolumbar cases. For example, a young Rottweiler with cervical SAD may suffer from a different condition than an older pug with a thoracolumbar SAD. Nevertheless, the presence of meningeal fibrosis in cervical SAD cases has been reported (137). This raises the question: is the presence of different stages of fibrosis and inflammation that leads to the progression of cases from a linear diverticulum to thick hyperplastic fibrotic arachnoid adhesions, which can ultimately result in a chronic inflammatory status (adhesive arachnoiditis)? Considering the difficulty in discerning these different stages with imaging and in identifying the primary origin of these conditions, further histopathological, proteomic, and genetic studies could shed light on the underlying causes of these diseases.

Challenges in treating different types of spinal meningeal conditions are encountered in both medical fields. While a young dog with SAD is comparable to SACs in humans, CM in old pugs appears more comparable to end-stage SAA. Further supporting this comparison, the presence of inflammatory changes, arachnoid epithelial hyperplasia, whorls of hyalinized collagen, and foci of calcification has been demonstrated in histopathological studies in old pugs (39, 58). This may reflect the poor outcome associated with CM in pugs (39, 58, 66). Since these conditions are uncommon, the possibility of sharing knowledge and building new approaches offers a unique opportunity for translational research. However, one of the main difficulties in studying these diseases is the different terminology used in the literature. While a disease is defined by the diagnostic criteria used, for the convenience of recording or discussion, a disease must have a name (138). Given the overlapping characteristics of spinal meningeal diseases in veterinary medicine (SAD and CM), the authors propose that this group of spinal meningeal pathologies is referred to as spinal meningeal adhesive diseases (SMADs). Given that in the majority of cases there is still uncertainty regarding the cause of these pathologies, SMADs could be further classified as idiopathic. Further research is necessary to assess whether these conditions stem from the same source. Even if this is not verified, this broader terminology acknowledges a wide range of interlinked conditions of the different spinal meningeal layers. If an underlying pathology is clearly identified, the authors propose that SMADs be further classified as secondary to the identified disease. The term ‘adhesive’ was chosen given that the presence of meningeal adhesions is a common factor among all these spinal meningeal pathologies in human and veterinary medicine. The term “adhesive” is more specific and less ambiguous. While the definitive origin of adhesions can only be speculated (inflammatory, congenital, or biomechanical), this addition allows for a clearer definition of these spinal meningeal conditions. Particularly in veterinary medicine, the term SMAD could be confused with conditions such as steroid-responsive meningitis arteritis. Idiopathic SCH could be separated from other spinal meningeal diseases, given its unique features. However, it was also included due to the difficulty in distinguishing idiopathic SCH from the other spinal meningeal diseases and the presence of adhesions, which are also seen with this condition, and the possible relation with intradural SAC being the precursor of this condition (77, 131–134). Furthermore, the term “idiopathic SMAD” would allow the exclusion of conditions such as congenital meningeal conditions (e.g., neural tube anomalies), diseases that secondarily affect the meninges, such as inflammatory diseases (e.g., immune-mediated or infectious meningomyelitis), and neoplastic pathologies (e.g., spinal meningiomas). A summary of the clinical and imaging features of spinal meningeal diseases that have been described in veterinary and human medicine can be found in Table 1. Finally, a future algorithm classification system for SMADs in veterinary medicine, based on key MRI features, surgical findings, and histopathological description of the meninges, could further enhance our understanding of these enigmatic pathologies.

## References

[1] Gage ED Hoerlein BF Bartels JE. Spinal cord compression resulting from a leptomeningeal cyst in the dog. J Am Vet Med Assoc. (1968) 152:1664–70. 5690014

[2] Hardie RJ Linn KA Rendano VT. Spinal meningeal cyst in a dog: a case report and literature review. J Am Anim Hosp Assoc. (1996) 32:477–80. doi: 10.5326/15473317-32-6-477, 8906723

[3] Parker AJ Smith CW. Meningeal cyst in a dog. J Am Anim Hosp Assoc. (1974) 10:595–7.

[4] Jurina K Grevel V. Spinal arachnoid pseudocysts in 10 rottweilers. J Small Anim Pract. (2004) 45:9–15. doi: 10.1111/j.1748-5827.2004.tb00188.x, 14756203

[5] McKee WM Renwick PW. Marsupialisation of an arachnoid cyst in a dog. J Small Anim Pract. (1994) 35:108–11. doi: 10.1111/J.1748-5827.1994.TB02550.X

[6] Oxley W Pink J. Amelioration of caudal thoracic syringohydromyelia following surgical management of an adjacent arachnoid cyst. J Small Anim Pract. (2012) 53:67–72. doi: 10.1111/J.1748-5827.2011.01146.X, 22122126

[7] Webb AA. Intradural spinal arachnoid cyst in a dog. Can Vet J. (1999) 40:506–8. 12001342 PMC1539774

[8] Skeen TM Olby NJ Muñana KR Sharp NJ. Spinal arachnoid cysts in 17 dogs. J Am Anim Hosp Assoc. (2003) 39:271–82. doi: 10.5326/0390271, 12755201

[9] F’son FO. Spinal arachnoid cyst in four dogs: diagnosis, surgical treatment and follow-up results. J Small Anim Pract. (1999) 40:544–9. doi: 10.1111/J.1748-5827.1999.TB03017.X, 10649600

[10] Gonçalves R Hammond G Penderis J. Imaging diagnosis: erroneous localization of spinal arachnoid cyst. Vet Radiol Ultrasound. (2008) 49:460–3. doi: 10.1111/J.1740-8261.2008.00408.X, 18833955

[11] Rohdin C Nyman HT Wohlsein P Hultin Jäderlund K. Cervical spinal intradural arachnoid cysts in related, young pugs. J Small Anim Pract. (2014) 55:229–34. doi: 10.1111/JSAP.12167, 24372140

[12] Bismuth C Ferrand FX Millet M Buttin P Fau D Cachon T . Original surgical treatment of thoracolumbar subarachnoid cysts in six chondrodystrophic dogs. Acta Vet Scand. (2014) 56:32. doi: 10.1186/1751-0147-56-32, 24884635 PMC4041341

[13] Gnirs K Ruel Y Blot S Begon D Rault D Delisle F . Spinal subarachnoid cysts in 13 dogs. Vet Radiol Ultrasound. (2003) 44:402–8. doi: 10.1111/J.1740-8261.2003.TB00476.X, 12939056

[14] Mól M Fernandes R Wheeler S Mariscoli M. Surgical outcomes of laminectomy, durotomy and a non-synthetic dura substitute application in ten dogs with a spinal subarachnoid diverticulum. Vet Sci. (2024) 11:128. doi: 10.3390/VETSCI11030128, 38535862 PMC10975197

[15] Hoey C Nye G Fadda A Bradshaw J Barker EN. Subarachnoid diverticulum associated with feline infectious peritonitis in a Siberian cat. J Feline Med Surg. (2020) 6:2055116920941477. doi: 10.1177/2055116920941477, 33149927 PMC7580156

[16] Ito D Ishikawa C Sekiguchi N Jeffery ND Kitagawa M. Utility of “MR myelography” in diagnosis of a presumed spinal subarachnoid diverticulum. J Small Anim Pract. (2020) 61:782. doi: 10.1111/jsap.13208, 33045762

[17] Lobacz MA Gutierrez Crespo BT Philbey AW Hammond G. Lumbar subarachnoid diverticulum secondary to a sarcoma in the sacral canal of a dog. Vet Rec Case Rep. (2015) 3:e000205. doi: 10.1136/VETRECCR-2015-000205

[18] Spinillo S Golini L Mariscoli M Motta L. Retrospective evaluation of surgical outcomes after closure of durotomy in eight dogs affected by spinal subarachnoid diverticulum. Open Vet J. (2020) 10:384–91. doi: 10.4314/OVJ.V10I4.5, 33614433 PMC7830178

[19] Smith CJ Guevar J. Spinal subarachnoid diverticula in dogs: a review. Can Vet J. (2020) 61:1162–9. 33149353 PMC7560765

[20] Jones B Behr S Shaw T Cappello R Jeffery N Liebel FX . Surgical techniques used in the management of intra-arachnoid diverticula in dogs across four referral centres and their immediate outcome. J Small Anim Pract. (2022) 63:520–5. doi: 10.1111/jsap.13486, 35137433 PMC9541676

[21] Lowrie ML Platt SR Garosi LS. Extramedullary spinal cysts in dogs. Vet Surg. (2014) 43:650–62. doi: 10.1111/j.1532-950X.2014.12200.x, 24798122

[22] De Frias JM Bhatti S De Decker S. An update on spinal arachnoid diverticula in dogs and cats. Vlaams Diergeneeskd Tijdschr. (2025) 94:125–45. doi: 10.21825/vdt.95429

[23] Mauler DA De Decker S De Risio L Volk HA Dennis R Gielen I . Signalment, clinical presentation, and diagnostic findings in 122 dogs with spinal arachnoid diverticula. J Vet Intern Med. (2014) 28:175–81. doi: 10.1111/jvim.12241, 24428321 PMC4895525

[24] De Frias JM Bhatti SFM Nye G Gonçalves R Harcourt-Brown T Fadda A . Spinal arachnoid diverticula in cats: clinical presentation, diagnostic imaging findings, treatment, and outcome. J Vet Intern Med. (2025) 39:e17294. doi: 10.1111/JVIM.17294, 39739353 PMC11683399

[25] Alisauskaite N Cizinauskas S Jeserevics J Rakauskas M Cherubini GB Anttila M . Short- and long-term outcome and magnetic resonance imaging findings after surgical treatment of thoracolumbar spinal arachnoid diverticula in 25 pugs. J Vet Intern Med. (2019) 33:1376–83. doi: 10.1111/JVIM.15470, 30844093 PMC6524397

[26] da Costa RC Cook LB. Cystic abnormalities of the spinal cord and vertebral column. Vet Clin North Am Small Anim Pract. (2016) 46:277–93. doi: 10.1016/j.cvsm.2015.10.010, 26706913

[27] Mauler DA De Decker S De Risio L Volk HA Dennis R Gielen I . Spinal arachnoid diverticula: outcome in 96 medically or surgically treated dogs. J Vet Intern Med. (2017) 31:849–53. doi: 10.1111/jvim.14714, 28426173 PMC5435043

[28] Shamir MH Shahar R Aizenberg I. Subarachnoid cyst in a cat. J Am Anim Hosp Assoc. (1997) 33:123–5. doi: 10.5326/15473317-33-2-123, 9111721

[29] Galloway AM Curtis NC Sommerlad SF Watt PR. Correlative imaging findings in seven dogs and one cat with spinal arachnoid cysts. Vet Radiol Ultrasound. (1999) 40:445–52. doi: 10.1111/J.1740-8261.1999.TB00373.X, 10528836

[30] Vignoli M Rossi F Sarli G. Spinal subarachnoid cyst in a cat. Vet Radiol Ultrasound. (1999) 40:116–9. doi: 10.1111/j.1740-8261.1999.tb01893.x, 10225519

[31] Schmidt MJ Schachenmayr W Thiel C Kramer M. Recurrent spinal arachnoid cyst in a cat. J Feline Med Surg. (2007) 9:509–13. doi: 10.1016/j.jfms.2007.04.006, 17618156 PMC10911502

[32] Sugiyama T Simpson DJ. Acquired arachnoid cyst in a cat: clinical review and case report. Aust Vet J. (2009) 87:296–300. doi: 10.1111/j.1751-0813.2009.00431.x, 19573158

[33] Pisoni L Cinti F Gallucci A Diana A Del Magno S Bellei E . Dura mater marsupialisation and outcome in a cat with a spinal subarachnoid pseudocyst: a case report. Veterinarni Med. (2014) 59:157–61. doi: 10.17221/7386-VETMED

[34] Adams RJ Garosi L Matiasek K Lowrie M. Acquired cervical spinal arachnoid diverticulum in a cat. J Small Anim Pract. (2015) 56:285–8. doi: 10.1111/jsap.12288, 25482364

[35] Hermans M Burger NC Kromhout K Bhatti SFM Cornelis I. Clinical and diagnostic findings in a dog and a cat with congenital hypothyroidism. Vet Rec Case Rep. (2020) 8:e001300. doi: 10.1136/VETRECCR-2020-001300

[36] Alcoverro E McConnell JF Sanchez-Masian D De Risio L De Decker S Gonçalves R. Late-onset recurrence of neurological deficits after surgery for spinal arachnoid diverticula. Vet Rec. (2018) 182:380–10. doi: 10.1136/VR.104579, 29288239

[37] Allison N Moeller RB. Spinal ataxia in a horse caused by an arachnoid diverticulum (cyst). J Vet Diagn Invest. (2000) 12:279–81. doi: 10.1177/104063870001200317, 10826847

[38] Fisher SC Shores A Simpson ST. Constrictive myelopathy secondary to hypoplasia or aplasia of the thoracolumbar caudal articular processes in pugs: 11 cases (1993–2009). J Am Vet Med Assoc. (2013) 242:223–9. doi: 10.2460/JAVMA.242.2.223, 23276100

[39] Rohdin C Ljungvall I Häggström J Leijon A Lindblad-Toh K Matiasek K . Thoracolumbar meningeal fibrosis in pugs. J Vet Intern Med. (2020) 34:797–807. doi: 10.1111/jvim.15716, 32003496 PMC7096664

[40] Driver CJ Rose J Tauro A Fernandes R Rusbridge C. Magnetic resonance image findings in pug dogs with thoracolumbar myelopathy and concurrent caudal articular process dysplasia. BMC Vet Res. (2019) 15:1–10. doi: 10.1186/S12917-019-1866-0, 31151444 PMC6544997

[41] Tauro A Rose J Rusbridge C Driver CJ. Surgical management of thoracolumbar myelopathies in pug dogs with concurrent articular facet dysplasia. Vet Comp Orthop Traumatol. (2019) 2:e60–72. doi: 10.1055/S-0039-1692147, 3785596

[42] Lourinho F Holdsworth A McConnell JF Gonçalves R Gutierrez-Quintana R Morales C . Clinical features and MRI characteristics of presumptive constrictive myelopathy in 27 pugs. Vet Radiol Ultrasound. (2020) 61:545–54. doi: 10.1111/VRU.12890, 32583954

[43] Flegel T Müller M-K Truar K Löffler C Oechtering G. Thoracolumbar spinal arachnoid diverticula in 5 pug dogs. Can Vet J. (2013) 54:969–73. 24155418 PMC3781430

[44] Chen AV Bagley RS West CL Gavin PR Tucker RL. Fecal incontinence and spinal cord abnormalities in seven dogs. J Am Vet Med Assoc. (2005) 227:1945–51. doi: 10.2460/JAVMA.2005.227.1945, 16379631

[45] Sadek AR Nader-Sepahi A. Spinal arachnoid cysts: presentation, management and pathophysiology. Clin Neurol Neurosurg. (2019) 180:87–96. doi: 10.1016/j.clineuro.2019.03.014, 30952036

[46] Liebel FX Platt S Matiasek K Houlton J Garosi L. Diagnosis and management of perineurial (Tarlov) cysts in two dogs. Vet Rec. (2013) 172:504. doi: 10.1136/VR.101213, 23542654

[47] Dyce J Heritage ME Houlton JEF Palmer AC. Canine spinal ‘arachnoid cysts’. J Small Anim Pract. (1991) 32:433–7. doi: 10.1111/J.1748-5827.1991.TB00980.X

[48] Hashizume CT. Cervical spinal arachnoid cyst in a dog. Can Vet J. (2000) 41:225–7. 10738602 PMC1476300

[49] Rylander H Lipsitz D Berry WL Sturges BK Vernau KM Dickinson PJ . Retrospective analysis of spinal arachnoid cysts in 14 dogs. J Vet Intern Med. (2002) 16:690–6. doi: 10.1111/J.1939-1676.2002.TB02409.X 12465766

[50] Sharon de Nies K Alexander Edwards R Bergknut N Beukers M Petrus Meij B. Caudal lumbar spinal cysts in two French bulldogs. Acta Vet Scand. (2018) 60:14. doi: 10.1186/s13028-018-0368-6, 29490674 PMC5831591

[51] Aikawa T Shimatsu T Miyazaki Y. Hemilaminectomy, diverticular marsupialization, and vertebral stabilization for thoracolumbar spinal arachnoid diverticula in five dogs. J Am Anim Hosp Assoc. (2019) 55:110–6. doi: 10.5326/JAAHA-MS-6762, 30776259

[52] Seiler GS Robertson ID Mai W Widmer WR Suran J Nemanic S . Usefulness of a half-fourier acquisition single-shot turbo spin-echo pulse sequence in identifying arachnoid diverticula in dogs. Vet Radiol Ultrasound. (2012) 53:157–61. doi: 10.1111/j.1740-8261.2011.01893.x, 22734150

[53] Tauro A Jovanovik J Driver CJ Rusbridge C. Clinical application of 3D-CISS MRI sequences for diagnosis and surgical planning of spinal arachnoid diverticula and adhesions in dogs. Vet Comp Orthop Traumatol. (2018) 31:083–94. doi: 10.3415/VCOT-16-12-0169, 29534275

[54] De Frias JM De Decker S De Stefani A Llabres-Diaz F. Description and clinical relevance of the variable conformation of canine spinal arachnoid diverticula. Vet Radiol Ultrasound. (2024) 65:344–51. doi: 10.1111/VRU.13365, 38572892

[55] Dewey CW Da Costa RC. "Chapter 13 – myelopathies: disorders of the spinal cord". In: Dewey CW Da Costa RC, editors. Practical Guide to Canine and Feline Neurology, 3rd Edn. Hoboken, New Jersey: John Wiley & Sons (2015). p. 370–1.

[56] Meren IL Chavera JA Alcott CJ Barker AK Jeffery ND. Shunt tube placement for amelioration of cerebrospinal fluid flow obstruction caused by spinal cord subarachnoid fibrosis in dogs. Vet Surg. (2017) 46:289–96. doi: 10.1111/VSU.12622, 28146294

[57] Gomes SA Targett M Mignan T Longo S James M Stee K . Post-surgical outcome and recurrence rates in thoracolumbar arachnoid diverticula undergoing durotomy alone or alongside a modified technique of subdural shunt-placement in dogs. Vet Surg. (2025) 54:972–82. doi: 10.1111/vsu.14236, 40018996

[58] Wachowiak IJ Patterson JS Winger KM Smiler KL Cole R Moon R . Thoracolumbar myelopathies in pug dogs. J Vet Intern Med. (2023) 37:618–25. doi: 10.1111/JVIM.16639, 36744714 PMC10061184

[59] Brander G Rohdin C Bianchi M Bergvall K Andersson G Ljungvall I . Multiple genetic loci associated with pug dog thoracolumbar myelopathy. Genes. (2023) 14:385. doi: 10.3390/GENES14020385, 36833311 PMC9957375

[60] Gallant CA. Constrictive myelopathy in an 11-year-old West Highland terrier dog. Can Vet J. (2020) 61:1319–21. 33299251 PMC7659884

[61] Bertram S ter Haar G De Decker S. Caudal articular process dysplasia of thoracic vertebrae in neurologically normal French bulldogs, English bulldogs, and pugs: prevalence and characteristics. Vet Radiol Ultrasound. (2018) 59:396–404. doi: 10.1111/VRU.12609, 29464823

[62] Nishida H Nakata K Maeda S Kamishina H. Prevalence and pattern of thoracolumbar caudal articular process anomalies and intervertebral disk herniations in pugs. J Vet Med Sci. (2019) 81:906–10. doi: 10.1292/JVMS.18-0521, 31092761 PMC6612495

[63] De Decker S Rohdin C Gutierrez-Quintana R. Vertebral and spinal malformations in small brachycephalic dog breeds: current knowledge and remaining questions. Vet J. (2024) 304:106095. doi: 10.1016/J.TVJL.2024.106095, 38458418

[64] Rohdin C Ljungvall I Jäderlund KH Svensson A Lindblad-Toh K Häggström J. Assessment of glial fibrillary acidic protein and anti-glial fibrillary acidic protein autoantibody concentrations and necrotising meningoencephalitis risk genotype in dogs with pug dog myelopathy. Vet Rec. (2024) 194:e3895. doi: 10.1002/vetr.3895, 38704817

[65] Greer KA Wong AK Liu H Famula TR Pedersen NC Ruhe A . Necrotizing meningoencephalitis of pug dogs associates with dog leukocyte antigen class II and resembles acute variant forms of multiple sclerosis. Tissue Antigens. (2010) 76:110–8. doi: 10.1111/j.1399-0039.2010.01484.x, 20403140

[66] Tauro A Driver CJ Rose J Fernandes R Rusbridge C. Case report: long-term surgical outcomes in pug dogs with articular facet dysplasia-associated thoracolumbar myelopathies. Front Vet Sci. (2025) 12:8444. doi: 10.3389/fvets.2025.1648444, 40919036 PMC12411195

[67] Capo G Calvanese F Tahhan N Creatura D Zaed I Bellina E . Prediction of MRI in intra-operative findings for spinal meningeal diseases. Neurochirurgie. (2025) 71:101661. doi: 10.1016/j.neuchi.2025.101661, 40057181

[68] Paramore CG. Dorsal arachnoid web with spinal cord compression: variant of an arachnoid cyst? Report of two cases. J Neurosurg. (2000) 93:287–90. doi: 10.3171/spi.2000.93.2.0287, 11012061

[69] Buntting CS Ham Y Teng KX Dimou J Gauden AJ Nair G. Scalpel sign: dorsal thoracic arachnoid web, thoracic arachnoid cyst and ventral cord herniation. Radiol. Case Reports. (2022) 17:3564–9. doi: 10.1016/j.radcr.2022.06.100, 35923346 PMC9340144

[70] Zhang D Papavassiliou E. Spinal intradural arachnoid webs causing spinal cord compression with inconclusive preoperative imaging: a report of 3 cases and a review of the literature. World Neurosurg. (2017) 99:251–8. doi: 10.1016/J.WNEU.2016.12.015, 27993741

[71] Voglis S Romagna A Germans MR Carreno I Stienen MN Henzi A . Spinal arachnoid web—a distinct entity of focal arachnopathy with favorable long-term outcome after surgical resection: analysis of a multicenter patient population. Spine. (2022) 22:126–35. doi: 10.1016/j.spinee.2021.06.018, 34175468

[72] Nisson PL Hussain I Härtl R Kim S Baaj AA. Arachnoid web of the spine: a systematic literature review. Spine. (2019) 31:175–84. doi: 10.3171/2019.1.SPINE181371, 31003220

[73] Shin JH Krishnaney AA. Idiopathic ventral spinal cord herniation: a rare presentation of tethered cord. Neurosurg Focus. (2010) 29:E10. doi: 10.3171/2010.3.FOCUS1089, 20593998

[74] Taylor TR Dineen R White B Jaspan T. The thoracic anterior spinal cord adhesion syndrome. Br J Radiol. (2012) 85:e123–9. doi: 10.1259/BJR/81458631, 22665931 PMC3474120

[75] Naggar A El Ouali I Aidi S Melhaoui A Ech-cherif el Kettani N Fikri M . Spinal arachnoid web: a systematic review of a rare entity, with two illustrative case reports. Egypt J Radiol Nucl Med. (2024) 55:178. doi: 10.1186/S43055-024-01348-2

[76] Bugdadi A Herbrecht A Aghakhani N Parker F. Clarifying rarity versus underreporting of idiopathic spinal arachnoid web: an analysis of the available evidence and the need for extended postoperative outcome reports. Surg Neurol Int. (2023) 14:367. doi: 10.25259/SNI_713_2023, 37941613 PMC10629311

[77] Zhang W Liu Z Wang K Zhang L Liu S Zhang X . Spinal adhesive arachnoidopathy, the disorder more than simply adhesive arachnoiditis: a comprehensive systematic review of 510 cases. CNS Neurosci Ther. (2024) 30:e70084. doi: 10.1111/cns.70084, 39435986 PMC11494685

[78] Kalsi P Hejrati N Charalampidis A Wu PH Schneider M Wilson JR . Spinal arachnoid cysts: a case series and systematic review of the literature. Brain Spine. (2022) 2:100904. doi: 10.1016/J.BAS.2022.100904, 36248116 PMC9560677

[79] Baig Mirza A Bartram J Vastani A Gebreyohanes A Al Banna Q Lavrador JP . Systematic review of surgical management of spinal intradural arachnoid cysts. World Neurosurg. (2022) 158:e298–309. doi: 10.1016/J.WNEU.2021.10.173 34728397

[80] Cuoco JA Muthukumar S Rogers CM Entwistle JJ Patel VM Olasunkanmi AL . Spinal intradural arachnoid cysts in adults: an institutional experience and literature review. Neurosurgery. (2023) 92:450–63. doi: 10.1227/NEU.0000000000002231, 36700689

[81] El-Hajj VG Singh A Pham K Edström E Elmi-Terander A Fletcher-Sandersjöö A. Long-term outcomes following surgical treatment of spinal arachnoid cysts: a population-based consecutive cohort study. Spine. (2023) 23:1869–76. doi: 10.1016/j.spinee.2023.08.011, 37604309

[82] Eroglu U Bozkurt M Kahilogullari G Dogan I Ozgural O Shah KJ . Surgical management of spinal arachnoid cysts in adults. World Neurosurg. (2019) 122:e1146–52. doi: 10.1016/j.wneu.2018.11.005, 30447456

[83] Fam MD Woodroffe RW Helland L Noeller J Dahdaleh NS Menezes AH . Spinal arachnoid cysts in adults: diagnosis and management. A single-center experience. Spine. (2018) 29:711–9. doi: 10.3171/2018.5.SPINE1820, 30265227

[84] Moses ZB Friedman GN Penn DL Solomon IH Chi JH. Intradural spinal arachnoid cyst resection: implications of duraplasty in a large case series. Spine. (2018) 28:548–54. doi: 10.3171/2017.8.SPINE17605, 29424675

[85] Funao H Nakamura M Hosogane N Watanabe K Tsuji T Ishii K . Surgical treatment of spinal extradural arachnoid cysts in the thoracolumbar spine. Neurosurgery. (2012) 71:278–84. doi: 10.1227/NEU.0b013e318257bf74, 22517249

[86] Tokmak M Ozek E Iplikcioglu AC. Spinal extradural arachnoid cysts: a series of 10 cases. J Neurol Surg Central Eur Neurosurg. (2015) 76:348–52. doi: 10.1055/s-0035-1547360, 26008955

[87] Klekamp J. A new classification for pathologies of spinal meninges, part 1: Dural cysts, dissections, and ectasias. Neurosurgery. (2017) 81:29–44. doi: 10.1093/NEUROS/NYX049, 28327939

[88] Klekamp J. A new classification for pathologies of spinal meninges, part 2: primary and secondary intradural arachnoid cysts. Neurosurgery. (2017) 81:217–29. doi: 10.1093/NEUROS/NYX050, 28327950

[89] Tarlov IM. Perineurial cysts of the spinal nerve roots. Arch Neurol Psychiatr. (1938) 40:1067–74. doi: 10.1001/ARCHNEURPSYC.1938.02270120017001

[90] Campeau NG Farnsworth PJ. Dorsal arachnoid web adjacent to paraspinal retained bullet: blunt post-traumatic etiology. Radiol Case Reports. (2024) 19:1254–7. doi: 10.1016/J.RADCR.2024.01.004, 38292796 PMC10825598

[91] Adib SD Schittenhelm J Kurucz P Hauser TK Tatagiba M. Surgical management of syringomyelia associated with spinal arachnoid web: strategies and outcomes. Neurosurg Rev. (2023) 46:152. doi: 10.1007/S10143-023-02071-8, 37358703 PMC10293323

[92] Alentado VJ Potts EA. Demonstration of ball valve mechanism of spinal arachnoid cyst expansion using intraoperative ultrasonography: 2-dimensional operative video. Operative Neurosurg. (2024) 26:106. doi: 10.1227/ons.0000000000000906, 37747347

[93] Fortuna A La Torre E Ciappetta P. Arachnoid diverticula: a unitary approach to spinal cysts communicating with the subarachnoid space. Acta Neurochir. (1977) 39:259–68. doi: 10.1007/BF01406736, 602855

[94] Teng P Rudner N. Multiple arachnoid diverticula. AMA Arch Neurol. (1960) 2:348–56. doi: 10.1001/archneur.1960.03840090112015, 13837415

[95] Perret G Green D Keller J. Diagnosis and treatment of intradural arachnoid cysts of the thoracic spine. Radiology. (1962) 79:425–9. doi: 10.1148/79.3.425, 14485487

[96] Fortuna A Mercuri S. Intradural spinal cysts. Acta Neurochir. (1983) 68:289–314. doi: 10.1007/BF01401186, 6880882

[97] Ramazanoglu AF Sarikaya C Varol E Aydin SO Etli MU Avci F . Surgical treatment of spinal arachnoid cysts: cyst excision or fenestration? Turk Neurosurg. (2022) 32:1002–6. doi: 10.5137/1019-5149.JTN.37597-22.2, 36066058

[98] Chatain GP Shrestha K Kortz MW Serva S Hosokawa P Ward RC . Impact of surgical timing on neurological outcomes for spinal arachnoid cyst: a single institution series. Neurospine. (2022) 19:453–62. doi: 10.14245/ns.2244130.065, 35793936 PMC9260545

[99] Kendall BE Valentine AR Keis B. Spinal arachnoid cysts: clinical and radiological correlation with prognosis. Neuroradiology. (1982) 22:225–34. doi: 10.1007/BF00342069, 7063114

[100] Ali HB Hamilton P Zygmunt S Yakoub KM. Spinal arachnoid web—a review article. J Spine Surg. (2018) 4:446–50. doi: 10.21037/jss.2018.05.08, 30069540 PMC6046336

[101] Smith M Ketterling M Gallaer A Kelner R Raps C Beaulieu AM. Spinal arachnoid web. Clin Pract Cases Emerg Med. (2024) 8:300–1. doi: 10.5811/cpcem.7189, 39158255 PMC11326078

[102] Yamamoto A Fujimoto M Aoki K Suzuki Y Mizuno M Suzuki H. A dorsal arachnoid EEB of the cervical spine: a case report. NMC Case Report J. (2021) 8:281–6. doi: 10.2176/NMCCRJ.CR.2020-0300, 35079476 PMC8769406

[103] Hines T Wang C Duttlinger C Thompson J Watford K Motley B . Thoracic dorsal arachnoid web with rapid onset of symptoms: a report of two cases and brief review of the literature. Surg Neurol Int. (2021) 12:323. doi: 10.25259/SNI_339_2021, 34345464 PMC8326092

[104] Hamilton P Bartley J Lawrence P Eisenring CV. Dorsal thoracic arachnoid web – confounders of a rare entity in the developing setting. Interdiscip Neurosurg. (2021) 25:101276. doi: 10.1016/j.inat.2021.101276

[105] Halloran PJ Leclair NK Bulsara KR Chozick BS Moss IL Becker KP . Dorsal arachnoid web of the thoracic spine associated with an arachnoid cyst and a presyrinx state as an unexplained cause of thoracic myelopathy: illustrative case. J Neurosurg Case Lessons. (2024) 8:CASE24313. doi: 10.3171/CASE24313, 39159494 PMC11337934

[106] Kawaguchi H Ono K Takabayashi N Ito T Harada K Sudo Y . Cine MRI is useful for the diagnosis of intradural arachnoid cyst with spinal arachnoid web: a case report. JBJS Case Connect. (2022) 12:818. doi: 10.2106/JBJS.CC.21.00818, 36040100

[107] Delgardo M Higgins D McCormick KL Reid P Canoll P McCormick PC. Clinical characteristics, outcomes, and pathology analysis in patients with dorsal arachnoid web. Neurosurgery. (2022) 90:581–7. doi: 10.1227/NEU.0000000000001884, 35290255

[108] Morrison T Datta R Masters L Boggild M Reddel S Brennan J. MR scalpel sign’ of spinal arachnoid web. Pract Neurol. (2022) 22:235–6. doi: 10.1136/practneurol-2021-003293, 35074799

[109] Reardon MA Raghavan P Carpenter-Bailey K Mukherjee S Smith JS Matsumoto JA . Dorsal thoracic arachnoid web and the “scalpel sign”: a distinct clinical-radiologic entity. Am J Neuroradiol. (2013) 34:1104–10. doi: 10.3174/ajnr.A3432, 23348759 PMC7964642

[110] Schultz R Steven A Wessell A Fischbein N Sansur CA Gandhi D . Differentiation of idiopathic spinal cord herniation from dorsal arachnoid webs on MRI and CT myelography. J Neurosurg Spine. (2017) 26:754–9. doi: 10.3171/2016.11.SPINE16696, 28338452

[111] Ruschel LG Agnoletto GJ Aurich LA Vosgerau RP. Dorsal arachnoid web and scalpel sign: a diagnostic imaging entity. Turk Neurosurg. (2018) 28:689–90. doi: 10.5137/1019-5149.JTN.18394-16.1, 27858382

[112] Brasil PM Pereira LP Távora DGF Camara ACF Macedo Filho CL Coimbra PPA. Imaging findings in dorsal thoracic arachnoid web and the differential diagnosis of “scalpel sign.”. Neurographics. (2020) 10:96–102. doi: 10.3174/ng.1900037

[113] Delamarter RB Ross JS Masaryk TJ Modic MT Bohlman HH. Diagnosis of lumbar arachnoiditis by magnetic resonance imaging. Spine. (1990) 15:304–10. doi: 10.1097/00007632-199004000-00011, 2353276

[114] Maulucci CM Ghobrial GM Oppenlander ME Flanders AE Vaccaro AR Harrop JS. Arachnoiditis ossificans: clinical series and review of the literature. Clin Neurol Neurosurg. (2014) 124:16–20. doi: 10.1016/J.CLINEURO.2014.06.024, 24999276

[115] Steel CJ Abrames EL O’Brien WT. Arachnoiditis ossificans – a rare cause of progressive myelopathy. Open Neuroimaging J. (2015) 9:13–20. doi: 10.2174/1874440001509010013, 26401174 PMC4578143

[116] Jurga S Szymańska-Adamcewicz O Wierzchołowski W Pilchowska-Ujma E Urbaniak Ł. Spinal adhesive arachnoiditis: three case reports and review of literature. Acta Neurol Belg. (2021) 121:47–53. doi: 10.1007/s13760-020-01431-1, 32833147 PMC7937595

[117] Anderson TL Morris JM Wald JT Kotsenas AL. Imaging appearance of advanced chronic adhesive arachnoiditis: a retrospective review. Am J Roentgenol. (2017) 209:648–55. doi: 10.2214/AJR.16.16704, 28639826

[118] Wright MH Denney LC. A comprehensive review of spinal arachnoiditis. Orthop Nurs. (2003) 22:215–9. doi: 10.1097/00006416-200305000-00010, 12803151

[119] Maillard J Batista S Medeiros F Farid G Maria PES Perret CM . Spinal adhesive arachnoiditis: a literature review. Cureus. (2023) 15:e33697. doi: 10.7759/cureus.33697, 36788823 PMC9922032

[120] Nadeem SF Baig AN Tariq QUA Shamim SM. Spinal arachnoiditis and syringomyelia: review of literature with emphasis on postinfectious inflammation and treatment. Surg Neurol Int. (2022) 13:299. doi: 10.25259/SNI_383_2022, 35928312 PMC9345109

[121] Hackert J Maßmann L Sure U Forsting M Kleinschnitz C Pul R . Immunotherapies in chronic adhesive arachnoiditis - a case series and literature review. eNeurologicalSci. (2021) 24:100350. doi: 10.1016/j.ensci.2021.100350, 34195394 PMC8225987

[122] Naito K Yamagata T Ohata K Takami T. Safety and efficacy of syringoperitoneal shunting with a programmable shunt valve for syringomyelia associated with extensive spinal adhesive arachnoiditis: technical note. World Neurosurg. (2019) 132:14–20. doi: 10.1016/j.wneu.2019.08.103, 31465850

[123] Shikata J Yamamuro T Iida H Sugimoto M. Surgical treatment for symptomatic spinal adhesive arachnoiditis. Spine. (1989) 14:870–5. doi: 10.1097/00007632-198908000-00018, 2781399

[124] Bhatia K Madhavan A Coutinho C Mathur S. Idiopathic spinal cord herniation. Clin Radiol. (2020) 75:721–9. doi: 10.1016/j.crad.2020.04.013, 32499121

[125] Iunes EA Barletta EA Suzuki FS Barba Belsuzarri TA de Araújo Paz D deCastro V . Idiopathic ventral spinal cord herniation: video report and systematic review. World Neurosurg. (2020) 139:592–602. doi: 10.1016/j.wneu.2020.04.190, 32376383

[126] Najjar MW Baeesa SS Lingawi SS. Idiopathic spinal cord herniation: a new theory of pathogenesis. Surg Neurol. (2004) 62:161–70. doi: 10.1016/j.surneu.2003.10.030, 15261515

[127] Neale N Ramayya A Welch W. Surgical management of idiopathic thoracic spinal cord herniation. World Neurosurg. (2019) 129:81–4. doi: 10.1016/j.wneu.2019.05.219, 31158530

[128] Sharma P Soin P Elbanan M Kochar PS. Understanding idiopathic spinal cord herniation – a comprehensive review of imaging and literature. J Clin Imaging Sci. (2019) 9:22. doi: 10.25259/JCIS-25-2019, 31448173 PMC6702865

[129] Harbert A Gilbert OE Franklin D Gibbs W Galgano M. Ventral spinal cord displacement: a guide to differentiating spinal cord herniation from dorsal arachnoid web with 2-dimensional operative video illustrations. Oper Neurosurg. (2025) 30:977–84. doi: 10.1227/ons.0000000000001724, 40778773

[130] Urbach H Kaden B Pechstein U Solymosi L. Herniation of the spinal cord 38 years after childhood trauma. Neuroradiology. (1996) 38:157–8. doi: 10.1007/BF00604806, 8692429

[131] Tekkök IH. Spontaneous spinal cord herniation: case report and review of the literature. Neurosurgery. (2000) 46:485–92. doi: 10.1097/00006123-200002000-00044. 10690740, 10690740

[132] Masuzawa H Nakayama H Shitara N Suzuki T. Spinal cord herniation into a congenital extradural arachnoid cyst causing Brown-Séquard syndrome: case report. J Neurosurg. (1981) 55:983–6. doi: 10.3171/jns.1981.55.6.0983, 7299475

[133] Isu T Iizuka T Iwasaki Y Nagashima M Akino M Abe H. Spinal cord herniation associated with an intradural spinal arachnoid cyst diagnosed by magnetic resonance imaging. Neurosurgery. (1991) 29:137–9. doi: 10.1097/00006123-199107000-00027, 1870677

[134] Sioutos P Arbit E Tsairis P Gargan R. Spontaneous thoracic spinal cord herniation: a case report. Spine. (1996) 21:1710–3. doi: 10.1097/00007632-199607150-00019, 8839477

[135] Summers J Balasubramani Y Chan P Rosenfeld J. Idiopathic spinal cord herniation: clinical review and report of three cases. Asian J Neurosurg. (2013) 8:97–105. doi: 10.4103/1793-5482.116386, 24049553 PMC3775190

[136] Groen RJM Middel B Meilof JF de Vos-van de Biezenbos JBM Enting RH Coppes MH . Operative treatment of anterior thoracic spinal cord herniation: three new cases and an individual patient data meta-analyisis of 126 case reports. Oper Neurosurg. (2009) 64:ons145–60. doi: 10.1227/01.NEU.0000327686.99072.E7 19240564

[137] de Lahunta A Glass E Kent M. “10 - small animal spinal cord disease”, In: Lahunta A de Glass E Kent M, editors. de Lahunta’s Veterinary Neuroanatomy and Clinical Neurology (5th Ed). Philadelphia: W.B. Saunders (2021). p.267–311.

[138] Moriyama IM. The classification of disease - a fundamental problem. J Chronic Dis. (1960) 11:462–70. doi: 10.1016/0021-9681(60)90011-4, 14424224

